# The role of micro health insurance in providing financial risk protection in developing countries- a systematic review

**DOI:** 10.1186/s12889-016-2937-9

**Published:** 2016-03-22

**Authors:** Shifa Salman Habib, Shagufta Perveen, Hussain Maqbool Ahmed Khuwaja

**Affiliations:** Department of Community Health Sciences, The Aga Khan University, Stadium Road, 74800 Karachi, Pakistan; School of Nursing & Midwifery, The Aga Khan University, Stadium Road, 74800 Karachi, Pakistan

**Keywords:** Mutual health insurance, Micro health insurance, Community based health insurance, Mutual health organizations, Developing countries, Financial protection, Pakistan, Systematic review

## Abstract

**Background:**

Out of pocket payments are the predominant method of financing healthcare in many developing countries, which can result in impoverishment and financial catastrophe for those affected. In 2010, WHO estimated that approximately 100 million people are pushed below the poverty line each year by payments for healthcare. Micro health insurance (MHI) has been used in some countries as means of risk pooling and reducing out of pocket health expenditure. A systematic review was conducted to assess the extent to which MHI has contributed to providing financial risk protection to low-income households in developing countries, and suggest how the findings can be applied in the Pakistani setting.

**Methods:**

We conducted a systematic search for published literature using the search terms “Community based health insurance AND developing countries”, “Micro health insurance AND developing countries”, “Mutual health insurance AND developing countries”, “mutual OR micro OR community based health insurance” “Health insurance AND impact AND poor” “Health insurance AND financial protection” and “mutual health organizations” on three databases, Pubmed, Google Scholar and Science Direct (Elsevier). Only those records that were published in the last ten years, in English language with their full texts available free of cost, were considered for inclusion in this review. Hand searching was carried out on the reference lists of the retrieved articles and webpages of international organizations like World Bank, World Health Organization and International Labour Organization.

**Results:**

Twenty-three articles were eligible for inclusion in this systematic review (14 from Asia and 9 from Africa). Our analysis shows that MHI, in the majority of cases, has been found to contribute to the financial protection of its beneficiaries, by reducing out of pocket health expenditure, catastrophic health expenditure, total health expenditure, household borrowings and poverty. MHI also had a positive safeguarding effect on household savings, assets and consumption patterns.

**Conclusion:**

Our review suggests that MHI, targeted at the low-income households and tailored to suit the cultural and geographical structures in the various areas of Pakistan, may contribute towards providing protection to the households from catastrophe and impoverishment resulting from health expenditures. This paper emphasizes the need for further research to fill the knowledge gap that exists about the impact of MHI, using robust study designs and impact indicators.

## Background

Financial catastrophe and impoverishment as a result of medical expenses, especially out of pocket (OOP) expenditures, has been a concern globally, more so, in developing countries [1] where the inadequacy of state provided health system results in alarmingly excessive OOP expenditure [2]. OOP payments for health care comprise 4-5.5 % of total household consumption in China, India, Bangladesh and Vietnam. This estimate is much lower, 1.4-2.7 % for Malaysia, Thailand, the Philippines, Sri Lanka and Hong Kong, as these countries are more economically stable than the first four countries, that are more heavily dependent on OOP spending for healthcare [3]. Literature suggests that approximately 100 million people are pushed below the poverty line each year by payments for health care [4].

The Alma-Ata declaration of 1978 advocated “health for all”, implying equitable access to health services for all individuals globally, regardless of socioeconomic class [5]. Despite this, unaffordability of healthcare is now accepted as one of the most decisive barriers to access to healthcare [6]. Substantial evidence from developing countries ascertain the continued presence of inequity in health, showing that the rich receive considerably more health benefits than the poor [7, 8]. More so, in the event of illness, many low-income households obtain sub-optimal care or forgo medical care altogether [9–11].

In Pakistan, the majority of health care is financed through OOP payments, which accounts for 55 % of the total healthcare costs [12]. Merely 26 % of the population is covered partially for its healthcare costs by the government, armed forces, corporate sector or other safety nets [13]. The gross total OOP health expenditures incurred by private households in the fiscal year 2011-12 amounted to PKR 315 billion ($ 2.9 billion) [12]. To cover the expenses associated with an event of sudden illness, the low-income households in Pakistan, often employ coping strategies such as drawing down savings, borrowing and selling productive assets such as cattle, poultry and land [14]. These coping mechanisms are frequently inadequate to cover the healthcare costs and the consequential debt may result in impoverishment of the effected household [9].

The health insurance strategy is gaining popularity, particularly in the developing countries, as a mode of providing financial protection from the healthcare costs. There are various diverse health insurance models operational in different countries, such as the national or social health insurance, which entails mandatory enrollment by the individuals [15] or voluntary insurance models such as private health insurance or micro health insurance (MHI).

The majority of the high-income countries rely heavily on general taxation (for example, the United Kingdom) or mandated health insurance (France, Germany) for healthcare financing [16]. On the contrary, in low-income countries, developing an efficient tax-funded health system maybe a difficult task, due to the dearth of a robust tax base and low institutional capacity to run the tax collecting apparatus efficiently [17]. Thus, in these countries, MHI may be able to provide financial protection, to a significant proportion of the population, against the downside of medical expenses. MHI is a kind of micro-insurance, which can be defined as “protection of low income people against financial risk, in exchange of payment of premiums, according to the probability and cost of the risk” [5, 18]

Varying terminologies, such as mutual health insurance or community-based health insurance, are used in different settings to designate MHI institutions. The three main defining criteria used in this paper for identifying any insurance program as MHI include a target population of low-income individuals or households, voluntary participation of the enrolled individuals or households and provision of health insurance services in exchange of premiums paid by the enrollees.

This paper aims to explore the prospect of instigation of a health financing reform in Pakistan with health insurance as a potential mode of providing financial security from the cost of healthcare consumption. We conducted a systematic review to provide cumulative evidence for the extent to which MHI has been useful, as an intervention in providing financial risk protection in developing countries, particularly to low-income households.

## Methods

### Search strategy

To conduct this systematic review, the principal investigator (SH) and co-investigator (SP) developed a search strategy to identify peer reviewed publications and reports of research studies and project evaluations in which MHI, in one of its organizational settings, was delivered to low income households within developing countries. We conducted a systematic search, from November and December 2015, using combinations of text words and thesaurus terms “Community based health insurance AND developing countries”, “Micro health insurance AND developing countries”, “Mutual health insurance AND developing countries”, “mutual OR micro OR community based health insurance" “Health insurance AND impact AND poor” “Health insurance AND financial protection” and “mutual health organizations”. These search terms were entered concurrently on three databases, Pubmed, Google Scholar and Science Direct (Elsevier). From these three databases, only those records, that were published in the last ten years having full texts that were available free of cost, were considered for inclusion in this review. As there was no funding available for conducting this systematic review, the authors decided to exclude non-English articles (to avoid translation costs). Only the abstract was available for one of the searched articles. The institutional librarian and the authors of the respective study were approached for the provision of full text.

**Fig. 1:**
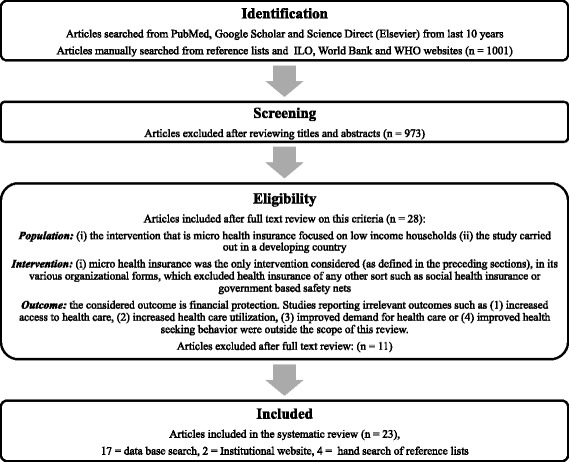
Systematic flow of search results

For Science Direct, further filters were applied to shortlist original articles, review articles and short communications for consideration, from journals pertaining to medicine and dentistry, nursing and allied and social sciences. The search was restricted to title only to limit the vast number of irrelevant articles from Google scholar, whereas for Science Direct and Pubmed, the search was limited to title and abstract. Other than peer reviewed journal articles, organizational research papers published by international bodies were also eligible for inclusion in this systematic review. Furthermore, hand searching was done on the reference list of the articles shortlisted by the above-mentioned strategy and those obtained from institutional websites such as World Bank, World Health Organization (WHO) and International Labor Organization (ILO), for relevant papers and reports. Figure 1 shows the flow of systematic search results.

The following inclusion criteria were considered for all publications and reports;

- Population: (i) the intervention, that is MHI, is delivered to low income households (ii) the study is carried out in a developing country
- Intervention: (i) micro health insurance was the only intervention considered (as defined in the preceding sections), in any of its organizational forms, which excluded health insurance of any other sort such as social health insurance or government based safety nets.
- Outcome: the considered outcome is financial protection. Studies reporting irrelevant outcomes such as (1) increased access to health care, (2) increased health care utilization, (3) improved demand for health care or (4) improved health-seeking behavior were outside the scope of this review.

### Data collection process

Our search resulted in 1001 papers from the three databases. After title and abstract review of these papers, 28 articles were shortlisted for review of full text in the light of our inclusion criterion. Out of these, 17 papers were identified as being eligible to be included in this systematic review.

Furthermore, two eligible studies from ILO’s website, and four from the reference lists of articles retrieved through systematic database search, were included in the final list of articles for data extraction, bringing the total number of included studies to 23. The first author independently assessed the title and abstracts and then reviewed the full texts of the shortlisted papers, in the light of the inclusion criteria, to determine whether those studies are eligible to be included in this review. Any ambiguity was resolved though discussion and consultation with the other two authors. Two authors extracted the data using a standard data extraction form (Table 1).

**Table 1:** Data extraction form

Title	
Journal/publication body	
Citation	
Year of publication	
Date of review	
Study design/evaluation design:	
Sampling technique (if given)	
WHO region	
Country/Area of intervention	
Type of health insurance	
Name of MHI or implementing body	
Other components of intervention (if any)	
Study population	
Data collection strategy?	
Objectives of the of study	
Measures of financial protection evaluated	
Key findings	

Data extracted from each article/report included the name of journal, publication date, WHO region and country of operation, study design, objectives of the study, data collection strategy, target population (low income households), measures of financial protection assessed and the key findings. In the final step, we assessed the adequacy and quality of information in the selected studies on the study design, sample size of the target population, sampling methods, interventions, evaluation methods and results. Finally, the information extracted from the 23 included studies was recorded in the standard data extraction form. Since the study population, organizational setting of health insurance, methodology, evaluation designs and measures of financial protection being evaluated in these studies were heterogeneous; we decided that it was not possible to conduct a meta-analysis or quantitative synthesis.

The list of excluded citations is presented in Table 2.

**Table 2:** Excluded citations with justification for exclusion

Justification for exclusion	Title of study	Author/Year
Insurance scheme not categorized as MHI	Effectiveness of public health insurance schemes on financial risk protection in Thailand: the assessments of purchasers’ capacities, contractors’ responses and impact on patients.	Vongmongkol V, Patcharanarumol W, Panichkriangkrai W, Pachanee K, Prakongsai P, Tangcharoensathien V, Hanson K, Mills A. (2011)
Other types of health insurance considered in creating impact	The Impact of Health Insurance Programs on Out-of-Pocket Expenditures in Indonesia: An Increase or a Decrease?	Aji B, De Allegri M, Souares A, Sauerborn R. (2013)
	Impact of Health Insurance on Health Care Treatment and Cost in Vietnam: A Health Capability Approach to Financial Protection	Nguyen KT, Khuat OT, Ma S, Pham DC, Khuat GT, Ruger JP. (2012)
	The effect of health insurance on financial protection and consumption smoothing: The case of Lebanon	Empirique É. The Effect of Health Insurance on Financial Protection and Consumption Smoothing: The Case of Lebanon. (2009)
Financial protection not entirely attributable to MHI	Do health sector reforms have their intended impacts? The World Bank’s Health VIII project in Gansu province, China-	Wagstaff A, Yu S. Do health sector reforms have their intended impacts? (2007)
No co-payments or premiums charged from the beneficiaries	The Impact of medical insurance for the poor in Georgia: a regression discontinuity approach	Bauhoff S, Hotchkiss DR, Smith O (2011)
	Promoting universal financial protection: health insurance for the poor in Georgia – a case study	Zoidze A, Rukhadze N, Chkhatarashvili K, Gotsadze G. (2013)
	An impact evaluation of medical insurance for poor in Georgia: preliminary results and policy implications	Gotsadze G, Zoidze A, Rukhadze N, Shengelia N, Chkhaidze N. (2015)
	Health insurance for the poor: impact on catastrophic and out-of-pocket health expenditures in Mexico	Galárraga O, Sosa-Rubí SG, Salinas-Rodríguez A, Sesma-Vázquez S. (2010)
Only abstract available	Does Health Insurance promote healthcare access and provide financial protection: empirical evidences from India	Kumar S. (2015)
Not classified as evaluation of impact of MHI on financial protection	Financial Protection in Health Insurance Schemes: A Comparative Analysis of Mediclaim Policy and CHAT Scheme in India	Vellakkal S. (2012)

### Quality assessment

The Mirza and Jenkins checklist was used to assess the quality of the articles meeting the inclusion criteria which includes: 1) Explicit study aims stated 2) Sample size justification given 3) Sample representative of population 4) Inclusion and exclusion criteria stated 5) Reliability and validity of measures justified 6) Response rate and dropout rate specified 7) Data adequately described 8) Statistical significance assessed 9) Discussion of generalizability given10) Null findings interpreted [19]. Only one study, out of the 23, gave complete details about the methods as per the checklist used (Table 3). However, due to scarcity of the available literature, the authors decided to include all the articles in this systematic review. Furthermore, the included studies were also categorized according to the outcomes they assessed and the quality of evidence, for each outcome, was classified as being high, moderate or low, based on the average of individual quality scores of the included studies (Tables 3 and 4). The quality of evidence for each of the studied outcomes varied between moderate and high (Table 4). The evidence found for the outcomes, reduction in CHE and protection of household savings, were particularly noted to be high in quality.

**Table 3:** Quality assessment of the included studies

Study	Explicit aims	Sample size justification or adequate	Justification sample representative of population	Inclusion and exclusion criteria stated	Reliability and validity of measures justified	Response rate and drop out specified	Data adequately described	Statistical significance assessed	Discussion of generalizability	Null findings interpreted	TOTAL
Hamid SA, Roberts J, Mosley P. Can micro health insurance reduce poverty? Evidence from Bangladesh. Journal of Risk and Insurance. 2011 Mar 1;78(1):57–82.	Y	Y	Y	Y	N	Y	Y	Y	Y	N	8
Yip W, Hsiao WC. Non-evidence-based policy: how effective is China's new cooperative medical scheme in reducing medical impoverishment? Social science & medicine. 2009 Jan 31;68(2):201–9.	Y	Y	Y	N	N	N	Y	Y	Y	N	6
Hou Z, Van de Poel E, Van Doorslaer E, Yu B, Meng Q. Effects of NCMS on access to care and financial protection in China. Health economics. 2014 Aug 1;23(8):917–34.	Y	Y	Y	Y	N	N	Y	Y	Y	N	7
Cheung D, Padieu Y. Heterogeneity of the effects of health insurance on household savings: Evidence from rural China. World Development. 2015 Feb 28;66:84–103.	Y	Y	Y	Y	Y	N	Y	Y	Y	Y	9
Sun Q, Liu X, Meng Q, Tang S, Yu B, Tolhurst R. Evaluating the financial protection of patients with chronic disease by health insurance in rural China. International Journal for Equity in Health. 2009;8:42. doi:10.1186/1475-9276-8-42.	Y	Y	Y	Y	Y	N	Y	Y	Y	N	8
Wagstaff A, Lindelow M, Jun G, Ling X, Juncheng Q. Extending health insurance to the rural population: An impact evaluation of China's new cooperative medical scheme. Journal of health economics. 2009 Jan 31;28(1):1–9.	N	Y	Y	Y	Y	Y	Y	Y	Y	N	8
Aggarwal A. Impact evaluation of India's ‘Yeshasvini’community-based health insurance programme. Health Economics. 2010 Sep 1;19(S1):5–35.	Y	Y	Y	Y	N	N	Y	Y	N	N	6
Savitha B, KB K. Microhealth insurance and the risk coping strategies for the management of illness in Karnataka: a case study. The International journal of health planning and management. 2013 Aug 1.	Y	Y	Y	Y	Y	N	Y	Y	Y	Y	9
Devadasan N, Criel B, Van Damme W, Ranson K, Van der Stuyft P. Indian community health insurance schemes provide partial protection against catastrophic health expenditure. BMC Health Services Research. 2007 Mar 15;7(1):43.	Y	Y	Y	Y	Y	N	Y	Y	Y	N	8
Wagstaff A. Health insurance for the poor: initial impacts of Vietnam's health care fund for the poor. World Bank Policy Research Working Paper. 2007 Feb 1(4134).	Y	Y	Y	N	Y	N	Y	Y	N	N	6
Wagstaff A. Estimating health insurance impacts under unobserved heterogeneity: the case of Vietnam's health care fund for the poor. Health economics. 2010 Feb 1;19(2):189–208.	Y	Y	Y	N	N	N	Y	Y	Y	N	6
Pham T, Pham TL. Does microinsurance help the poor? Evidence from the targeted health microinsurance program in Vietnam 2004–2008. International Labor Organization. 2012 Feb. Research paper No. 11	Y	Y	Y	Y	Y	Y	Y	Y	Y	Y	10
Alkenbrack S, Lindelow M. The Impact of Community‐Based Health Insurance on Utilization and Out‐of‐Pocket Expenditures in Lao People's Democratic Republic. Health economics. 2013 Dec 1.	Y	Y	Y	Y	Y	Y	Y	Y	Y	N	9
Bodhisane S, Pongpanich S. The Impact of Community Based Health Insurance in Enhancing Better Accessibility and Lowering the Chance of Having Financial Catastrophe Due to Health Service Utilization A Case Study of Savannakhet Province, Laos. International Journal of Health Services. 2015 Jul 20:0020731415595609.	Y	Y	N	Y	N	N	Y	Y	N	N	5
Franco LM, Diop FP, Burgert CR, Kelley AG, Makinen M, Simpara CH. Effects of mutual health organizations on use of priority health-care services in urban and rural Mali: a case–control study. Bulletin of the World Health Organization. 2008 Nov;86(11):830–8.	Y	Y	N	Y	N	N	Y	Y	N	N	5
Dercon S, Gunning JW, Zeitlin A, Lombardini S. The impact of a health insurance programme: Evidence from a randomized controlled trial in Kenya. Research Paper. 2012 Nov(24).	Y	Y	Y	Y	Y	N	Y	Y	N	N	7
Parmar D, Reinhold S, Souares A, Savadogo G, Sauerborn R. Does Community-Based Health Insurance Protect Household Assets? Evidence from Rural Africa. Health services research. 2012 Apr 1;47(2):819–39.	Y	Y	Y	Y	Y	Y	Y	Y	N	N	8
Haddad S, Ridde V, Yacoubou I, Mák G, Gbetié M. An evaluation of the outcomes of mutual health organizations in Benin.	Y	Y	N	Y	Y	N	Y	Y	N	N	6
Saksena P, Antunes AF, Xu K, Musango L, Carrin G. Mutual health insurance in Rwanda: evidence on access to care and financial risk protection. Health policy. 2011 Mar 31;99(3):203–9.	Y	Y	Y	N	N	N	Y	Y	Y	Y	7
Lu C, Chin B, Lewandowski JL, Basinga P, Hirschhorn LR, Hill K, Murray M, Binagwaho A. Towards universal health coverage: an evaluation of Rwanda Mutuelles in its first eight years. PLoS One. 2012 Jun 1;7(6):e39282.	Y	Y	Y	Y	Y	N	Y	Y	Y	Y	9
Kihaule A. Impact of Micro Health Insurance Plans on Protecting Households Against Catastrophic Health Spending in Tanzania. GSTF Journal of Nursing and Health Care (JNHC). 2015 Aug 27;2(2).	Y	Y	Y	Y	N	N	N	Y	Y	N	6
Dekker M, Wilms A. Health Insurance and Other Risk-Coping Strategies in Uganda: The Case of Microcare Insurance Ltd. World Development. 2010 Mar 31;38(3):369–78.	Y	N	N	N	N	N	Y	Y	Y	N	4
Chankova S, Sulzbach S, Diop F. Impact of mutual health organizations: evidence from West Africa. Health policy and planning. 2008 Jul 1;23(4):264–76.	Y	Y	Y	Y	N	N	Y	Y	N	N	6

**Table 4:** Summary of findings

Outcome (measure of financial protection)	Relative effect	Number of studies and participants	Quality of evidence (Quality score)	Comments
Reduction in OOP expenditure	Not estimable	13 studies	Moderate quality (Quality score 6.8)	The effect size is not quantifiable as the results in the majority of studies are not presented statistically. Individuals (or households where individuals count is not available) covered by the study may show the most widely studied outcomes in terms of individuals/households covered. The quality of evidence has been classified as high, moderate and low by taking the average of the individual study scores for the various outcomes (refer to Table 3)
		Individuals covered: 202.615 (12 studies)		
		Households covered: 2974 (1 study)		
Reduction in CHE	Not estimable	7 studies	High quality (Quality Score 7.9)	
		Individuals covered: 82448 (5 studies)		
		Households covered: 3226 (2 studies)		
Reduction in total health expenditures	Not estimable	3 studies	Moderate quality (Quality score 6.8)	
		Individuals covered: 51599 (3 studies)		
Reduction in poverty	Not estimable	2 studies	Moderate quality (Quality score 7.0)	
		Households covered: 5709 (2 studies)		
Improvement in consumption patterns	Not estimable	1 study	Moderate quality (Quality score 7.0)	
		Individuals covered: 145		
Protection of household assets	Not estimable	4 studies	Moderate quality (Quality score 6.8)	
		Individuals covered: 43499 (3 studies)		
		Households covered: 890 (1 study)		
Protection of household savings	Not estimable	3 studies	High quality (Quality score 8.0)	
		Individuals covered: 26591 (2 studies)		
		Households covered: 1312 (1 study)		
Reduction in household borrowings	Not estimable	4 studies	Moderate quality (Quality score 7.3)	
		Individuals covered: 43644(4 studies)		

Quality of evidence criteria: score of ≤5 is low; score of ˃5 and ≤7.5 is moderate; and score of ≥7.6 is high

## Results

As described above, from the list of 28 articles, originally shortlisted after title/abstract review, 11 were excluded either due to lack of evidence on financial protection being directly attributed to MHI, the health insurance scheme not meeting the definition of MHI in terms of premiums paid by the insured or in terms of voluntary participation, or the effect being evaluated in the paper being the impact on health center costs and not the enrolled households.

Table 5 provides a snapshot of the 23 articles that were finally included, summarizing name of the MHI project (or the implementing organization), study design and setting, the measure of financial protection assessed and the key findings. The quality assessment of the included articles is presented in Table 3.

**Table 5:** Methodological details and key findings of the included studies

S. No.	Citation	WHO region	Objective of the study	Country/Target population	Name of MHI Scheme	Study design, sampling technique, evaluation design	Measure of financial protection	Key findings/Outcomes
1	Hamid SA, Roberts J, Mosley P. Can micro health insurance reduce poverty? Evidence from Bangladesh. Journal of Risk and Insurance. 2011 Mar 1;78(1):57–82.	Asia	To assess whether the addition of MHI to the microcredit programs of GB has an effect on poverty	Bangladesh/Poor households	Grameen Bank MHI	Cross sectional, Multistage sampling, Comparison between program and control areas	Poverty 1. Household income, 2. Household non income assets, 3. Food sufficiency 4. Probability of being above or below the poverty line	Positive association found between MHI and household income, ownership of assets, food sufficiency and poverty reduction. Result was statistically significant for food sufficiency only
2	Yip W, Hsiao WC. Non-evidence-based policy: how effective is China's new cooperative medical scheme in reducing medical impoverishment? Social science & medicine. 2009 Jan 31;68(2):201–9.		To assess the effectiveness of the NCMS model in reducing medical impoverishment	China/Rural population	New Cooperative Medical Scheme (NCMS)	Comparison study, Convenience sampling, Comparison between two study (insurance) groups	Poverty	NCMS reduced poverty headcount by 3.5-3.9 % The RMHC would reduce poverty by 8.3-13.1 %
3	Hou Z, Van de Poel E, Van Doorslaer E, Yu B, Meng Q. Effects of NCMS on access to care and financial protection in China. Health economics. 2014 Aug 1;23(8):917-34		To identify the impact of NCMS on access to care and financial protection by exploiting the variation in NCMS design across counties.			Cross sectional, Simple random sampling, Calculation of scheme generosity based on (i) the copayment; (ii) the reimbursement rate; and (iii) the ceiling.	OOP expenditure	No effects found on spending in the full sample, but conditional upon use, NCMS reduces the share of OOP spending for an outpatient visit and increases OOP spending per inpatient stay (among users) Total spending per hospitalization had increased (among users)
4	Cheung D, Padieu Y. Heterogeneity of the effects of health insurance on household savings: Evidence from rural China. World Development. 2015 Feb 28;66:84–103.		To explore the heterogeneity of the impact of NCMS on household savings across income groups in rural China.			Cross sectional Purposive sampling, Comparison between income quartiles, Propensity Score Matching	Household savings	Higher middle-income participants deplete their savings significantly compared to non-participant households. This difference suggests a decrease in savings secondary to reduction of household patrimony. Higher middle-income participants save less than non-participants. There was no impact of the health care scheme on the poorest and richest households.
5	Sun Q, Liu X, Meng Q, Tang S, Yu B, Tolhurst R. Evaluating the financial protection of patients with chronic disease by health insurance in rural China. International Journal for Equity in Health. 2009;8:42. doi:10.1186/1475-9276-8-42.		To investigate the extent to which patients suffering from chronic disease in rural China face catastrophic expenditure on healthcare, and how far the New Co-operative Medical Insurance Scheme (NCMS) offers them financial protection against this.	China/Rural households with chronic illness patients		Cross Sectional, Multistage sampling: County: Township: Village: Household, Comparison between insured and non insured	CHE	Between 8 and 11 % of non-NCMS members and 13 % of NCMS members did not seek any medical care for chronic illness. A greater proportion of NCMS members in the poorest quintile faced CHE as compared to those in the richest quintile Overall a slightly higher proportion of non-NCMS members than NCMS member households faced CHE but the difference was not statistically significant.
6	Wagstaff A, Lindelow M, Jun G, Ling X, Juncheng Q. Extending health insurance to the rural population: An impact evaluation of China's new cooperative medical scheme. Journal of health economics. 2009 Jan 31;28(1):1–9.		To assess the impacts on township health centers and county hospitals in all 189 counties. To investigate the issue of how the characteristics of different NCMS schemes—their generosity and which services are reimbursable—affect their impact.	China/Rural households		Cross Sectional, Multi-stage stratified random sampling, Comparison between insured and non insured, Propensity score matching	OOP CHE	The overall household OOP spending on health care does not appear to have been reduced by NCMS. Cost of delivery was reduced by NCMS. Cost of OPD was not reduced. NCMS appears to have resulted in people receiving more expensive health care per visit.
7	Aggarwal A. Impact evaluation of India's ‘Yeshasvini’community-based health insurance programme. Health Economics. 2010 Sep 1;19(S1):5–35./		To evaluate the impact of India’s Yeshasvini community-based health insurance programme on health-care utilization, financial protection, treatment outcomes and economic well-being.	India/Cooperative rural farmers and informal sector workers	Yeshasvini	Cross sectional, Multi stage random sampling, Comparison between intervention and control groups, Propensity score matching	Borrowing Sale of assets Household Savings Overall health expenditures	Total borrowings are 36 % and 30 % less for enrollees. The payments made out of savings, incomes, and other sources, on the other hand, are up to 74 % less for enrollees. Borrowings and/or asset sales associated with primary health-care use are 61 % lower for the relatively worse-off group among the insured. Overall health expenditures are 19–20 % higher for YH enrollees compared with uninsured cooperatives
8	Savitha B, KB K. Microhealth insurance and the risk coping strategies for the management of illness in Karnataka: a case study. The International journal of health planning and management. 2013 Aug 1./		To evaluate the impact of SampoornaSuraksha Program, on risk coping strategies of households faced with medical illness in Karnataka state, India	India/Rural population	Sampoorna Suraksha	Cross sectional descriptive, Multistage cluster sampling	Borrowing Household savings Sale of assets;	A lower percentage of insured individuals (57.2 %) relied on borrowing compared with newly insured (79.5 %) or uninsured individuals (75.2 %) (p < 0.05). Insured individuals used more savings (32.7 %) than newly insured (24.7 %) (p > 0.05). Sale of assets was found to be high in insured group than in newly insured but lower than that in uninsured groups (p > 0.05). The odds of the incidence of borrowing increased by a factor of 4.636 in newly insured and by a factor of 6.407 in uninsured compared with the insured individual
9	Devadasan N, Criel B, Van Damme W, Ranson K, Van der Stuyft P. Indian community health insurance schemes provide partial protection against catastrophic health expenditure. BMC Health Services Research. 2007 Mar 15;7(1):43		To determine whether insured households are protected from catastrophic health expenditure (CHE)	India/ACCORD_ rural population SEWA- women informal workers	ACCORD & SEWA	Cross sectional, Desk review of claims register, Comparison between two health insurance schemes	OOP expenditure CHE	67 % of ACCORD and 34 % of SEWA members protected from OOP payments 8 % (currently at 3.5 %) at ACCORD and 49 % at SEWA (currently 23 %) would have experienced CHE in the absence of an insurance scheme.
10	Wagstaff A. Health insurance for the poor: initial impacts of Vietnam's health care fund for the poor. World Bank Policy Research Working Paper. 2007 Feb 1(4134).		To estimate the impact of HCFP by comparing out-of-pocket payments and utilization between those covered by HCFP and comparable individuals not covered.	Vietnam/Poor households, households in poor localities, minorities	Health Care Fund for the Poor (HCFP)	Cross Sectional, Comparison between of insured and uninsured, Propensity Score Matching	OOP expenditure	HCFP reduces the risk of catastrophic OOP spending. There was no perceptible impact on (average) OOP spending,
11	Wagstaff A. Estimating health insurance impacts under unobserved heterogeneity: the case of Vietnam's health care fund for the poor. Health economics. 2010 Feb 1;19(2):189–208.		To estimate the impact of Vietnam’s health insurance program for poor households (health care fund for the poor, or HCFP) in a way that is robust to the biases introduced by unobserved heterogeneity.			Cross Sectional, Comparison between insured and uninsured, Triple differencing with matching	OOP expenditure	HCFP appears to have reduced OOP spending on health care considerably, A significant impact on OOP spending is not evident in a single difference, i.e. comparing spending in 2006 across the treated and control groups. It is evident in a double difference – i.e. comparing the 2004–2006 change across the two groups
12	Pham T, Pham TL. Does microinsurance help the poor? Evidence from the targeted health microinsurance program in Vietnam 2004–2008. International Labor Organization. 2012 Feb. Research paper No. 11		To assess whether HCFP program improves health care seeking behavior of the poor with respect to access to health care, OOP spending, and preventive care behavior;			Cross sectional, Stratified random cluster sampling, Impact Evaluation: using impact measures of Intention to treat effect, and treatment effect of the treated	OOP expenditure CHE	MHI reduced the OOP health care expenditure of poor participants, through a price reduction effect. propensity of having a CHE is lowered by 19 % among insured
13	Alkenbrack S, Lindelow M. The Impact of Community‐Based Health Insurance on Utilization and Out‐of‐Pocket Expenditures in Lao People's Democratic Republic. Health economics. 2013 Dec 1		To estimate the MHI program’s impact on utilization and out-of-pocket expenditures	Lao PDR/Informal workers	CBHI implemented by MoH	Cross sectional, two-stage cluster sampling, Comparison between insured and uninsured, Propensity Score matching	Health expenditures CHE	CBHI members’ total payments, conditional on any use, were less than those of the uninsured ($62.71 for CBHI versus $98.70 for non-CBHI members). 14.7 % of insured inpatient service users live in households with CHE compared with 27.4 % of uninsured inpatient users
14	Bodhisane S, Pongpanich S. The Impact of Community Based Health Insurance in Enhancing Better Accessibility and Lowering the Chance of Having Financial Catastrophe Due to Health Service Utilization A Case Study of Savannakhet Province, Laos. International Journal of Health Services. 2015 Jul 20:0020731415595609		To determine the role of community-based health insurance in making health care services accessible and in preventing financial catastrophe resulting from personal payment for inpatient services.	Lao PDR/Informal sector		Cross sectional, simple random sampling , Comparison between insured and uninsured	CHE	There was no difference in terms of probability of financial catastrophe from health service utilization between insured and uninsured households. Insurance status does not significantly improve accessibility and financial protection against CHE
15	Franco LM, Diop FP, Burgert CR, Kelley AG, Makinen M, Simpara CH. Effects of mutual health organizations on use of priority health-care services in urban and rural Mali: a case–control study. Bulletin of the World Health Organization. 2008 Nov;86(11):830–8./	Africa	To examine the effects of a community-based mutual health organization (MHO) on utilization of priority health services, financial protection of its members and inclusion of the poor and other target groups.	Mali/Informal sector	4 MHOs	Case control, Simple random sampling, Desk review, Comparison between member and non-member households	Health expenditures OOP expenditure	Lower household health expenditures as a percentage of overall cash consumption and lower OOP payments for fever treatments were reported among the insured. Health expenditure out of total cash is 5.6 to 6.4 in MHO members and 6.2 to 8.9 % in non members
16	Dercon S, Gunning JW, Zeitlin A, Lombardini S. The impact of a health insurance programme: Evidence from a randomized controlled trial in Kenya. Research Paper. 2012 Nov(24)./		To investigate the impact of Bimaya Jamali health insurance on health care utilization and health care outcomes, and a variety of outcomes not directly related to health.	Kenya/Informal sector/tea farmers	Wananchi Savings and Credit Cooperative Society/Bimaya Jamali	Randomized Controlled Trial	Health expenditures Borrowing Household consumption	Positive impact of MHI was reported on 1. Net health expenditures 2. Informal borrowing for medical costs 3. Food consumption 4. Non-food consumption 5. Overall consumption
17	Parmar D, Reinhold S, Souares A, Savadogo G, Sauerborn R. Does Community-Based Health Insurance Protect Household Assets? Evidence from Rural Africa. Health services research. 2012 Apr 1;47(2):819–39./		To evaluate whether community-based health insurance (CBHI) protects household assets in rural Burkina Faso, Africa	Burkina Faso/Rural population	Assurance Maladie à Base Communautaire	Randomized controlled trial Random sampling	Household assets	MHI seemed to protect and increase household assets 7 % increase in 2006 and 16 % increase in 2007 was recorded.
18	Haddad S, Ridde V, Yacoubou I, Mák G, Gbetié M. An evaluation of the outcomes of mutual health organizations in Benin.		To evaluate the benefits attributable to membership in a mutual health organization in a rural region of Benin.	Benin/Rural low income households	10 MHOs	Cross sectional, Purposive and convenience sampling; Document review, Comparison between intervention and control groups	OOP	MHI significantly reduced hospitalization expenses among members. Particular benefits to the poor were not proven.
19	Saksena P, Antunes AF, Xu K, Musango L, Carrin G. Mutual health insurance in Rwanda: evidence on access to care and financial risk protection. Health policy. 2011 Mar 31;99(3):203–9./		To examine the effect of mutual health insurance (MHI) on utilization of health services and financial risk protection.	Rwanda/Mainly informal sector	Not mentioned	Cross sectional, Comparison between insured and uninsured	OOP expenditure Financial burden	Insured households spent significantly less OOP: only 3.5 % of their CTP compared to 6.6 % for non-insured households. Households insured with MHI had a lower financial burden, with only 20.1 % of them spending over 10 % compared to 41.6 % for non-insured.
20	Lu C, Chin B, Lewandowski JL, Basinga P, Hirschhorn LR, Hill K, Murray M, Binagwaho A. Towards universal health coverage: an evaluation of Rwanda Mutuelles in its first eight years. PLoS One. 2012 Jun 1;7(6):e39282.		To evaluate the impact of Mutuelles on achieving universal coverage of medical services and financial risk protection in its first eight years of implementation	Rwanda/General Population Children Under 5 Years Pregnant women	Mutelles	Cross sectional, Comparison between insured and uninsured, Propensity Score matching	OOP expenditure CHE	The average annual household OOP spending for insured was significantly lower (5,744 RWF) than that of the uninsured households (8,755 RWF). The percentage of the insured households with CHE (5.1 %) was significantly lower than that (10.5 %) of uninsured households.
21	Kihaule A. Impact of Micro Health Insurance Plans on Protecting Households Against Catastrophic Health Spending in Tanzania. GSTF Journal of Nursing and Health Care (JNHC). 2015 Aug 27;2(2		To analyze whether households’ membership in micro health insurance funds provide them with the protection against catastrophic health spending, when sick.	Tanzania/Rural and urban population	Not mentioned	Cross sectional, Comparison between insured and uninsured	CHE OOP expenditure	Insured households were protected against CHE during episodes of illness Reduction in OOP expenditure among the members was reported
22	Dekker M, Wilms A. Health Insurance and Other Risk-Coping Strategies in Uganda: The Case of Microcare Insurance Ltd. World Development. 2010 Mar 31;38(3):369–78.		To explore the relationship between health insurance and other risk-coping strategies used to finance medical expenditures in Uganda.	Uganda/ Formal and informal sector (study restricted to rural, informal sector population)	Microcare insurance	Cross sectional, Convenience sampling	OOP expenditure Sale of assets Borrowing	OOP expenditures on health care were significantly higher in the uninsured households: USh 186,640 (US$ 100.88) in last 12 months compared to the insured households USh 83,420 (US$ 45.09). 44 % of the uninsured households and 56 % of those insured had enough cash to pay for health care. Uninsured households sold assets worth USh 138,940 (US$ 75.10) while insured households sold USh 35,030 (US$ 18.94) worth of assets. OOP expenditure per illness was USh 31,252 (US$ 16.89) lower for insured households. Insured borrowed less money per illness (a reduction of USh 42,828 or US$ 23.15).
23	Chankova S, Sulzbach S, Diop F. Impact of mutual health organizations: evidence from West Africa. Health policy and planning. 2008 Jul 1;23(4):264–76.		To add to the limited evidence on the impact of MHOs on utilization and out-of-pocket payments.	Ghana, Mali, Senegal/Households registered and not registered in 3 study sides serving as cases and comparison groups	Ghana: 1 MHO: Nkoranza Health Insurance Scheme Mali: 4 MHOs: Bougoulaville, Wayerma, Kemeni, Blaville Senegal: 27 MHOs—all MHOs in Thies region that had been operational in the 2 years preceding the study	Cross sectional, Comparison between insured and insured households	OOP expenditure	In Ghana, hospital OOP expenditure averaged US$2 among insured, compared with US$44 for non-beneficiaries. In Senegal, inpatient OOP expenditures was US$61 for MHO members, US$234 for non-members. There was no difference in OOP expenditures for outpatient care between MHO members and non-members in Ghana, Mali and Senegal

## A: Characteristics of the included studies

### Study setting and target population

Out of the 23 studies/reports included in this review, five are from China, all reporting the financial protection provided by the New Cooperative Medical Scheme (NCMS) in China. NCMS is operated, and heavily subsidized, by the government of China and provides voluntary insurance for its poor rural population. It was initiated in 2003, and was primarily aimed at covering catastrophic health expenditures (CHE). One of these studies [20] conducted a survey of 354 counties in the Western and Central regions of China. The second [21] and the third studies [22] used data from 6 counties in the two provinces of Ningxia and Shandong. The fourth study [23] used data from the China Health and Nutrition survey conducted in 333 counties where NCMS was implemented. The final study from China reports results of the impact evaluation of NCMS from 189 counties [24]. Three of the included studies are from India, reporting the financial protection provided by four different MHI programs. From these, the Yeshaswini program and the Sampoorna Suraksha Program were both functional in the state of Karnataka and targeted at informal sector workers and general rural population respectively [25, 26]. SEWA was being operated in Gujarat, with informal female workers and ACCORD in the rural regions of Tamil Nadu [27]. The study on the Yeshaswini program (Aggarwal 2010) was conducted in 82 villages across 16 districts of the state whereas the evaluation of the Sampoorna Suraksha program (Savitha 2013) was conducted in 10 randomly selected taluks across three districts of the state. Nine of the included studies were based on MHI institutions that were operational in Africa; two from Rwanda, one each from Kenya, Tanzania, Uganda, Burkina Faso, Benin and Mali and a cross country study evaluating MHI in Ghana, Mali and Senegal. The two studies from Rwanda used data from an Integrated Living Conditions Survey (ILCS) and ILCS and Rwanda Demographic Health Survey respectively in the 12 provinces of Rwanda [28, 29]. The study from Kenya was conducted across 150 tea centres in Nyeri District of Kenya [30]. The Tanzania study used data from Tanzania Demographic and Health Survey conducted nationwide across 10,300 households, involving men and women aged 15-49 years [31]. In Uganda the evaluation of the Micro care’s health insurance scheme was obtained from informal workers in the rural area of Kisiizi and urban centre of Kampala [32]. The study from rural Africa (Parmar 2012) was based on data from 42 villages and 1 town of Burkina Faso [33]. The study from West Africa (Chankova S, Sulzbach S, Diop 2008) was based on three individual studies conducted in the rural district of Nkoranza and Offinso in Ghana, rural district of Bla and urban commune of Sikasso in Mali, and the Thies region in Senegal [34]. Of the two projects grounded in Lao PDR, one was conducted in 87 villages across six districts, encompassing three provinces and the other was conducted in the province of Savannkhet [35, 36]. In Benin, the evaluation was carried out in a rural zone in the country’s Central and Northern areas, in a territory served by 10 MHOs [37]. In Bangladesh, the study was conducted in Madhabpur, Joy Mantap (in district Manikgonj) and Pakutia (in district Tangail) branches of Grameen Bank MHI [38]. The three studies from Vietnam report the measures of financial protection among the beneficiaries of Vietnam’s Health Care Fund for the Poor, using data from a series of household surveys (Vietnam Households Living Standard Surveys) conducted in all provinces (rural and urban) of the country [39–41].

### Study designs

Out of the 23 studies, 20 studies used cross sectional study design. Only two studies, conducted in Kenya and Burkina Faso, used a randomized control trial study design [30], while the one study conducted in Mali was a case control study [34]. Two of the cross sectional studies, used the cross sectional data to compare two different MHI schemes while 16 cross-sectional studies were based on a comparison between insured and uninsured. From these 16 cross sectional studies, six used the technique of propensity score matching and one study [40] used triple difference with matching to match the characteristics of the two comparison groups (insured and uninsured), in order to mitigate possible selection bias that could have occurred.

### Measures of financial protection and outcomes

Finally, the analysis of the 23 studies included in the review, resulted in the identification of following measures of financial protection. 1) OOP 2) CHE 3) Total health expenditures 4) Poverty 5) Consumption patterns 6) Household assets 7) Household savings (Table 4).

#### Out of pocket expenditure

Thirteen out of the 23 included studies have used reduced OOP expenditure on health as the measure for assessing the financial protection provided by MHI.

Studies from Tanzania, Uganda and Benin, done to evaluate effects of MHI by comparing insured versus un-insured households, showed that members of the health insurance scheme incur much less OOP, at the point of service, when seeking health care. In Uganda, the uninsured households, had spent USh 186,640 (US$ 100.88) on average, in the last 12 months, compared to the households insured by Microcare insurance, who had spent a much lower amount of USh 83,420 (US$ 45.09) [32].

In India, it was found that 67 % of insured households at ACCORD MHI scheme and 34 % of insured households at SEWA MHI scheme were protected from making OOP payments for healthcare [27].

In Vietnam, participation in the Health Care Fund for the Poor (HCFP) showed differential effects on the OOP health expenditure of poor participants at different stages of the scheme. The World Bank Policy Research Paper of 2007, showed that HCFP had no impact on OOP, and the poor end up spending a significant proportion of their incomes on healthcare [39]. In 2010, Wagstaff [40] again stated that a significant impact on OOP expenditure could only be proven in a double difference, that is comparing the 2004–2006 change across the treatment and the control groups, but not in a single difference (just the difference in spending in a single year across the 2 groups). The ILO research paper (2012) however, showed a significant reduction in OOP expenditure among the insured, through a price reduction effect [41].

A study by Saksena et al in Rwanda revealed that MHI enrollment contributes towards diminishing financial burden on the enrolled households. The study defined financial burden as ratio of OOP expenditure to capacity-to-pay (CTP). The households insured with MHI scheme spent only 3.5 % OOP out of their total CTP whereas the uninsured households spent 6.6 %. Only 20.1 % of the insured households spent beyond 10 % of their CTP compared to 41.6 % of the non-insured [28].

On the contrary, few studies depict that MHI has only been partially effective in reducing OOP or CHE. In China, NCMS was launched to alleviate enrollees’ monetary burden and shield from medical impoverishment. A study done to evaluate the impact of the NCMS scheme in rural China, found that NCMS reduces the OOP expenditure at the outpatient level but raises it at the inpatient level alongside total spending per episode of hospitalization [21]. Similar findings were reported by another study where it was concluded that even though cost of deliveries was reduced by NCMS, the OOP expenditure on OPD and other inpatient services did not drop [24].

A case control study from Mali [42] conducted to study the impact of membership in four MHOs showed that there was reduced OOP expenditure among the insured for fever treatments only. Similarly, a study conducted in three West African countries of Ghana, Mali and Senegal showed decline in OOP among the insured for hospitalization only [34]. In Ghana, the cost of an event of hospitalization varied significantly between beneficiaries and non-beneficiaries, which was $2 and $44 respectively. In Senegal this amount was $61 for beneficiaries and $235 for non-beneficiaries. In these countries, MHO membership did not appear to have a significant effect on OOP expenditures for curative outpatient care as the expenses incurred were almost the same for members and non-members [34].

#### Catastrophic health expenditures

Our results are suggestive of a likely association between MHI and CHE. In Tanzania, MHI was proven to be providing financial protection to the member households against CHE during episodes of illness [with a CHE co-efficient of -0.0686 (p = 0.04) for the poor households and -0.08015 (p = 0.022 for the non-poor households)], where CHE had been defined as OOP spending on health care that exceeds 25 percent of the total household budget [31].

A study from India revealed that in the absence of community based health insurance, 8 % of the households insured with ACCORD MHI scheme and 49 % with SEWA MHI scheme would have faced CHE [27].

In Vietnam also, a study reveals that HCFP MHI program, helped reduce the incidence of CHE when dealing with adverse health events, by increasing the overall health awareness among the beneficiaries that encouraged them to go for more regular medical checkups [39].

The second included study from Rwanda, studying the effect of Mutelles, a mutual health organization proved that the proportion of the insured households with CHE (5.1 percent) was considerably lower than that (10.5 percent) of uninsured households with CHE [29]. Similar findings were reported from Lao PDR, where it was found that 14.7 % of insured inpatient service users live in households with CHE compared with 27.4 % of uninsured inpatient users [36]. In contrast, another study done on the members of the same CBHI scheme in Lao PDR, but in a different province of the country, showed that there was no difference in the probability of financial catastrophe from health expenditures between insured and uninsured households [35].

In China, a study showed that among the households with chronic diseased patients, slightly higher proportion of non-NCMS member households than NCMS member households faced catastrophic expenditure, but the difference was not statistically significant [22].

#### Total health expenditures

The study conducted in west African country of Mali, showed that the MHO membership was associated with lower household health expenditures as a percentage of overall cash consumption [42].

In contrast, the paper from India, based on the Yeshasvini CBHI shows that the overall health expenditures were 19-20 % higher among the insured than the uninsured [25].

In some settings, despite of MHI reducing the overall health expenditures, it may not be able to relieve the poorest segments of the society from financial burden. The impact evaluation study from Benin, included in our paper, indicated that even though the hospitalization expenses were significantly reduced among members of the MHO, the overall benefits of the scheme for the poor were not proven [37].

#### Poverty

A research paper, also included in this review, evaluates the effect of Grameen Bank MHI on poverty reduction, in the rural regions of Bangladesh. The indicators used in this survey to gauge poverty included household income, non-income assets, food sufficiency and probability of being above or below the poverty line. The results of the impact assessment showed a positive relation between MHI and the mentioned indicators, hence proving that MHI plays a role in poverty reduction. However, the results were statistically significant for only food sufficiency [38]. A paper from China studies the role of government-led NCMS in preventing medical impoverishment among the rural population by doing a comparison between NCMS beneficiaries and beneficiaries of another MHI scheme RMHC. It was found that NCMS reduced poverty headcount by 3.5-3.9 %. The authors also proved that the RMHC, in contrast, could reduce poverty by 8.3-13.1 % [20].

#### Consumption patterns and household assets

A randomized control trial from ILO’s micro-innovation facility in Kenya, describes the impact of Wananchi Savings and Credit Cooperative Society, a micro insurance scheme [30]. It was found that enrollment in the MHI component of the scheme, reduced net health expenditures, decreased informal borrowings for medical costs and improved non-food and overall consumption. Also, the households covered with MHI were found to have greater values of assets and savings.

Another study underlined the impact of a community-based health insurance (CBHI) scheme, named Assurance Maladie à Base Communautaire’, on household assets in rural Burkina Faso, Africa, through a randomized community based trial [33]. The results indicated that this CBHI scheme, not only shielded the household assets but also increased them in Nouana Health District. The per capita household assets increased by 7 % in 2006 and by 16 % in 2007.

A study from India, evaluating the role of the ‘Yeshasvini’ CBHI scheme, found that the sale of assets for obtaining primary healthcare is 61 % lower for the CBHI beneficiaries belonging to the poorer segment [25].

#### Household savings

Three studies were found to highlight the effect of MHI scheme on household savings.

The study on India’s CBHI ‘Yeshaswini’ program, states that the use of household savings to pay for healthcare is up to 74 % less among the insured than the uninsured [25]. The paper researching the effect of Sampoorna Suraksha program in India indicated that a larger percent of the uninsured group (35.3 %) used household savings to pay for healthcare, compared to insured (32.7 %) and newly insured (24.7 %) groups. However, this result was insignificant (p > 0.05) [26].

The study from rural China gave results, which were in contrast of the other included studies. This study examines the effect of the New Cooperative Medical Scheme (NCMS) on household savings across income quartiles in rural China. It was found that NCMS has a negative bearing on savings of middle-income participants; the lower-middle income participants deplete their savings rapidly while the higher-middle income save less compared to the non-participants. This scheme has not proven to have any influence on the household savings of the poorest households [20].

#### Household borrowing

Two studies, both from India, discuss the effect of MHI on household borrowings as a risk coping strategy. Among the members of the Yeshasvini CBHI scheme in India, the total borrowings were 30-36 % less than the non-enrollees [25]. Whereas among the enrollees of Sampoorna Suraksha program in Karnataka, India it was found that a lower percentage of insured individuals (57.2 %) relied on borrowings compared to the newly insured (79.5 %) or uninsured individuals (75.2 %) (p < 0.05) [26].

The Table 4 provides a summary of findings (SoF), using guidelines from the Cochrane Handbook for Systematic Review of Interventions version 5.1.0 (modified for the purpose of systematic review) [43]. For the outcomes presented in this review, the effect size is non-quantifiable as the majority of studies included, have not presented the findings statistically, but have just observed the overall qualitative effect of the intervention (MHI) on the outcome (measures of financial protection).

The SoF table also illustrates the number of individuals (or the households, where the individuals are not mentioned) being covered in the included studies. This gives an overall picture of the most widely studied outcomes of MHI.

We have also commented on the overall quality of evidence accumulated for the various outcomes. The Mirza and Jenkins checklist, (Table 3) has been used to categorize the quality of overall evidence as low, moderate or high, by taking average of the individual quality scores of the included studies (Table 4).

## Discussion

The findings from the systematic review indicate that largely, MHI has had a positive bearing on the financial protection of low-income households in developing countries.

To the best of our knowledge, this study is the first systematic review that broadly examines the role of MHI in providing financial protection in developing countries.

Our review also points out that no impact evaluation has been undertaken for a vast majority of MHI schemes, even though currently more than 100 such schemes are operational in various developing countries like India [44] and Sub Saharan Africa [43]. In Africa alone, over 14 million (about 1.8 %) people, mostly those working in the informal sector, are covered by an MHI scheme [45].

Furthermore, in many papers, the effect of MHI being considered is other than financial protection, such as change in healthcare utilization, health seeking behavior or healthcare access. Despite this, the available evidence clearly demonstrates that MHI, in several ways, can contribute in provision of financial protection against the cost of healthcare consumption, particularly to low-income households in developing countries. In this section, we have also deliberated upon the applicability of MHI schemes in Pakistan as an alternative to user fees as a health financing mechanism.

Findings from 11 out of 23 studies included in this review, point towards reduction in OOP expenditure as a direct result of usage of MHI. This finding is in line with the vision of WHO, that has advocated for the implementation of prepaid health financing mechanisms for mitigating the detrimental effects of user fees [46]. OOP spending in Pakistan, as percent of the gross domestic product (GDP) is 55 % [12]. Even when attending the government-funded health care system, a patient is expected to cover various costs like user fees as well as medication, transportation and other consumables [13]. Globally, high OOP spending has been associated with impoverishment or financial catastrophe [47] making it vital to consider a risk pooling mechanism, such as MHI, to cover health financing needs of a country like Pakistan, where 21.04 % of the population live below the poverty line of $1.25 per day [12, 48].

In this review, we found convincing evidence regarding the positive effect of MHI on risk coping strategies such as the sale of assets, depletion of household savings, decreased consumption patterns and household borrowings. In Pakistan, the coping strategies most often employed by low-income households to address healthcare costs, include drawing down savings, borrowing and selling productive assets such as cattle, poultry and land [14]. These are frequently inadequate to cover the health expenditure and the consequential debt can result in the impoverishment of the effected households [9].

We could find very little evidence on the direct impact of MHI on poverty. Therefore, no conclusion can be drawn in this regard. However the reduction in OOP expenditure, borrowings and sale of assets could all indirectly prevent impoverishment due to health expenditures [9]. In addition to financial protection, MHI schemes have also shown to improve access to and utilization of health care in many developing countries [48, 49]. The improved health status can lead to higher economic growth [50], which may also, in turn, lead to poverty reduction.

In light of the evidence presented in this review, there is a strong rationale for the wide-ranging implementation of MHI in Pakistan, specifically in areas of the country where supply of health services may not be deficient. However, feasible MHI schemes in developing economies may be arduous to develop and challenging to sustain [51]. The threats to sustainability include high expense ratios, higher probabilities of loss because of poorer health statuses of the insured; inadequate infrastructure; risky living and working environments; and lack of understanding of the insurance mechanism [51]. In these markets, the MHI designs are kept simple due to data limitations, high transaction costs and low levels of education of the people and therefore may suffer from adverse selection [51].

In Pakistan, the insurance industry is still in infancy. In 2005, Pakistan was ranked at 58th position in the global insurance market by volume, with China and India at 9^th^ and 15^th^ positions respectively [52]. In the Asian region, the share of insurance density for Pakistan is 5 % as opposed to 18 % of Sri Lanka, 32 % of China and 43 % of India [53]. Insurance density provides evidence on the expenditure on insurance provision and is calculated as ratio of premiums collected per capita [54]. In 2008, insurance penetration in Pakistan was merely 0.7 % of the GDP [51]. Within this industry, there is a limited share of micro insurance [51]. Only a few MHI schemes have been operational in Pakistan, and none have been subjected to impact evaluation. A qualitative study rolled out in Ghizar district of Pakistan, to study the effect of MHI implemented by the Aga Khan Agency for Microfinance (AKAM) [55] revealed an extremely low first year penetration of 4 %, mostly due to cost constraints, lack of awareness about the availability of the product, lack of understanding of the product mechanism or trust in the scheme [55]. The AKAM scheme has also been associated with adverse selection primarily due to lack of individual risk classification [55]. There is a certain need to take steps to counter adverse selection to ensure business feasibility. Secondly, the schemes currently being offered primarily cover catastrophic expenditure, excluding preexisting conditions, outpatient visits, outpatient medication and maternity services because of which the uptake remains low [56]. Offering customized packages to the consumers, after a careful market survey could help improve uptake. Furthermore, marketing remains a crucial strategy to attain good enrollment rates for voluntary insurance schemes such as MHI [55].

The measures of financial protection assessed in this review are suggestive of a likely association between MHI and financial protection. However, while considering the applicability of MHI in Pakistan, the policy makers must be cognizant of the barriers to feasibility of implementation. The findings of this review could be used, with careful consideration of supply side and feasibility issues, in forming the basis for any MHI scheme in the country.

The MOOSE checklist was utilized to report this systematic review, which can be considered a strength of this study (Table 6) [57].

**Table 6:** MOOSE Checklist

Item No	Recommendation	Reported on Page No
Reporting of background should include		
1	Problem definition	2
2	Hypothesis statement	2
3	Description of study outcome(s)	2
4	Type of exposure or intervention used	2
5	Type of study designs used	2
6	Study population	2
Reporting of search strategy should include		
7	Qualifications of searchers (eg, librarians and investigators)	2
8	Search strategy, including time period included in the synthesis and key words	2
9	Effort to include all available studies, including contact with authors	3
10	Databases and registries searched	2-3
11	Search software used, name and version, including special features used (eg, explosion)	Not applicable. No search software was used
12	Use of hand searching (eg, reference lists of obtained articles)	3
13	List of citations located and those excluded, including justification	Table 2 (page 5) and Table 5 (page 10–17)
14	Method of addressing articles published in languages other than English	3
15	Method of handling abstracts and unpublished studies	3
16	Description of any contact with authors	3
Reporting of methods should include		
17	Description of relevance or appropriateness of studies assembled for assessing the hypothesis to be tested	3
18	Rationale for the selection and coding of data (eg, sound clinical principles or convenience)	3. No coding of data was required
19	Documentation of how data were classified and coded (eg, multiple raters, blinding and interrater reliability)	4 No coding of data was required
20	Assessment of confounding (eg, comparability of cases and controls in studies where appropriate)	9 (matching of cases and controls done in few included studies)
21	Assessment of study quality, including blinding of quality assessors, stratification or regression on possible predictors of study results	4
22	Assessment of heterogeneity	Not applicable
23	Description of statistical methods (eg, complete description of fixed or random effects models, justification of whether the chosen models account for predictors of study results, dose-response models, or cumulative meta-analysis) in sufficient detail to be replicated	Not applicable
24	Provision of appropriate tables and graphics	Tables given
Reporting of results should include		
25	Graphic summarizing individual study estimates and overall estimate	Not applicable
26	Table giving descriptive information for each study included	Table 5 (page 10–17)
27	Results of sensitivity testing (eg, subgroup analysis)	Not applicable
28	Indication of statistical uncertainty of findings	Not applicable
Reporting of discussion should include		
29	Quantitative assessment of bias (eg, publication bias)	Not applicable. This is a qualitative systematic review
30	Justification for exclusion (eg, exclusion of non-English language citations)	3 & 5 (Table 2)
31	Assessment of quality of included studies	6-8 (Table 3), 22
Reporting of conclusions should include		
32	Consideration of alternative explanations for observed results	22
33	Generalization of the conclusions (ie, appropriate for the data presented and within the domain of the literature review)	22
34	Guidelines for future research	22
35	Disclosure of funding source	Not applicable

*From*: Stroup DF, Berlin JA, Morton SC, et. al., for the Meta-analysis Of Observational Studies in Epidemiology (MOOSE) Group. Meta-analysis of Observational Studies in Epidemiology. A Proposal for Reporting. JAMA. 2000;283(15):2008–2012. doi: 10.1001/jama.283.15.2008

Transcribed from the original paper within the NEUROSURGERY® Editorial Office, Atlanta, GA, United Sates. August 2012

### Limitations

Our review points to a narrow evidence base as only 23 studies could meet our inclusion criteria. The review also revealed a large variation in study design and quality. Only two studies had used experimental study design, while most others used observational analysis to compare various measures of financial protection among insured individuals with those in a control group (perhaps the same individuals but formerly insured, or different individuals without prior insurance). Only one study met all the quality criteria used in this review.

This systematic review emphasizes the potential usefulness of developing guidelines for appropriately measuring the impact of MHI. There is a definite need to conduct studies using more rigorous designs and impact indicators for MHI impact evaluations. It is also imperative to measure its effect on other aspects of coping strategies for health expenditures such as foregone care or decreased economic productivity.

## Conclusion

Micro health insurance, targeted at low-income households in developing countries, has made a considerable contribution in providing financial protection from health care expenditures. The results indicated a positive influence of MHI on OOP, CHE, poverty, health expenditures, household consumption, borrowings, sale of assets and household savings (protective effect).

The findings in this review can be used, with careful deliberation, to guide future policies and MHI programmes in Pakistan. However, the upcoming schemes should be tailored to suit the cultural and sociopolitical environments, specific to various geographical settings in Pakistan. There is also a dire need to conduct more research in this field. With the use of more robust study designs and impact indicators, the emerging evidence will be increasingly significant.

## Abbreviations

CHE: catastrophic health expenditure
CTP: capacity to pay
FATA: Federally Administered Tribal Areas
GDP: gross domestic product
HCFP: health care fund for the poor
ILO: International Labour Organization
KPK: Khyber Pakhtunkhwa
MHI: micro health insurance
MHO: mutual health organization
NCMS: new cooperative medical scheme
NHA: National Health Accounts
OOP: out of pocket
URBI: urban resident basic medical insurance
WHO: World Health Organization
